# Molecular Dynamics Simulation on the Influences of Nanostructure Shape, Interfacial Adhesion Energy, and Mold Insert Material on the Demolding Process of Micro-Injection Molding

**DOI:** 10.3390/polym11101573

**Published:** 2019-09-27

**Authors:** Jin Yang, Can Weng, Jun Lai, Tao Ding, Hao Wang

**Affiliations:** 1 College of Mechanical and Electrical Engineering, Central South University, Changsha 410083, China; yj20141116@csu.edu.cn (J.Y.); 15827170706@163.com (J.L.); dingtao@csu.edu.cn (T.D.); 2 State Key Laboratory of High Performance Complex Manufacturing, Central South University, Changsha 410083, China; 3 Department of Mechanical Engineering, Faculty of Engineering, National University of Singapore, 9 Engineering Drive 1, Singapore 117575, Singapore; mpewhao@nus.edu.sg

**Keywords:** interfacial adhesion energy, nanostructure, demolding, micro-injection molding

## Abstract

In micro-injection molding, the interaction between the polymer and the mold insert has an important effect on demolding quality of nanostructure. An all-atom molecular dynamics simulation method was performed to study the effect of nanostructure shape, interfacial adhesion energy, and mold insert material on demolding quality of nanostructures. The deformation behaviors of nanostructures were analyzed by calculating the non-bonded interaction energies, the density distributions, the radii of gyration, the potential energies, and the snapshots of the demolding stage. The nanostructure shape had a direct impact on demolding quality. When the contact areas were the same, the nanostructure shape did not affect the non-bonded interaction energy at PP-Ni interface. During the demolding process, the radii of gyration of molecular chains were greatly increased, and the overall density was decreased significantly. After assuming that the mold insert surface was coated with an anti-stick coating, the surface burrs, the necking, and the stretching of nanostructures were significantly reduced after demolding. The deformation of nanostructures in the Ni and Cu mold inserts were more serious than that of the Al_2_O_3_ and Si mold inserts. In general, this study would provide theoretical guidance for the design of nanostructure shape and the selection of mold insert material.

## 1. Introduction

In recent years, micro/nanostructured products have been applied and developed in many fields, such as biomedical, aerospace, clean energy, and electronic communication [1,2,3,4]. Micro-injection molding technology, due to its low cost and high efficiency, is regarded as one of the most promising manufacturing methods for micro/nanostructures. It mainly includes four stages as the injection, the packing, the cooling, and the demolding [5,6]. Among them, the demolding stage is crucial for the high-precision replication of nanostructured parts, which directly affects the function and characteristics of the products [7,8,9].

The common demolding defects of nanostructures are bending, fracture, surface burr, necking [10,11], etc. Most of the defects are significantly caused by the adhesion and friction of the polymer-mold insert interface [9,12]. In order to reduce the adhesion between the mold insert and the polymer, many effective methods have been developed. For example, the coating technologies on the surface of a mold insert, like ceramic coating, fluoropolymer coating, self-assembled monolayer (SAM) coating and diamond-like carbon coating were used to decrease the surface roughness and the friction coefficient [6,8,10,13,14]. Otherwise, the interfacial adhesion energy can be controlled by changing the melt and the mold temperature. The adhesion strength was found to increase with the increases of the melt and the mold temperatures in the micro-injection molding. However, when the melt and the mold temperatures were higher than a limit, the adhesion strength would be reduced [15].

In the demolding process, it is necessary to investigate the adhesion force at the polymer-mold insert interface to reduce the deformation and improve the forming quality of the nanostructured parts. However, the experimental studies can only analyze the influences of processing parameters on the replication quality during the process [16], and cannot further understand the molding and demolding mechanism of polymers. Therefore, numerical simulation methods are introduced by researchers to analyze the deformation behavior of polymers and the interfacial adhesion properties. Some scholars have used the finite element method (FEM) based on the continuum theory to describe the friction force, the adhesion force and the thermal stress caused by shrinkage during the demolding process [17,18,19]. However, the friction and adhesion forces on nanostructures are mainly determined by the non-bonded interaction energy of the atoms at the polymer-mold insert interface when the size of the nanostructure is in the range of nanoscale [20].

The molecular dynamics (MD) simulation method can accurately analyze the interaction mechanism of the polymer-mold insert interface from the molecular and atomic level in the micro-injection molding process. This method complements the mechanism gap that is difficult to describe by the continuum theory. At present, the MD method has been widely applied to most of the research areas, such as the nanoimprint lithography technology [21,22,23], the micro-injection molding technology [24,25,26], the direct injection joining technology [27,28], the calculation of interfacial adhesion [29,30], and the simulation of self-assembled monolayer [31]. For the study of polymer molding, the MD method was mainly used by researchers to analyze the influences of nanostructure shapes [22,23,27,32], the mold insert and polymer materials [30,33], the anti-stick treatment of surface [32,34], the processing parameters [26,32], and other factors on the molding qualities. However, the above studies mainly focus on the nanoimprint lithography technology, and there are few reports on the micro-injection molding. It is very important to investigate the non-bonded interaction at the polymer-mold insert interface to reduce the deformation of nanostructure and improve the forming quality. Although the scale of micro-injection molding of MD simulation is greatly different from that of the experiments, it can still provide valuable theoretical guidance for the experiments.

In this study, the MD method was utilized to simulate the demolding process of micro-injection molding for nanostructures with different contact interfaces. The influences of nanostructure shapes, interfacial adhesion energy, and mold insert materials on molding qualities were investigated. The defects in nanostructures could be observed clearly during the demolding process. Based on these simulation results, the density distributions, potential energies, and radii of gyration of nanostructures during the demolding process were calculated to explain its deformation behaviors. Furthermore, the effects of non-bonded interaction at different contact interfaces on the deformation of nanostructures were compared.

## 2. Materials and Methods

### 2.1. Model Construction

**Figure 1:**
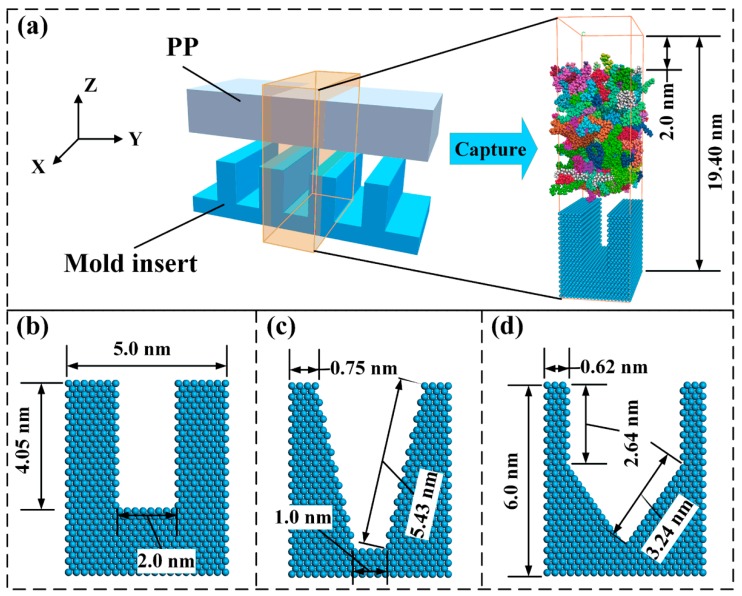
Initial simulation model and mold inserts with nanocavity for different shapes: (**a**) Definition of the simulation model, (**b**) rectangular shape, (**c**) trapezoidal shape, (**d**) tapered shape.

In this study, the all-atom model was used for the MD simulation. Considering the computational performance and time constraints, only a single nanostructure was selected in the simulation. The model was consisted of the upper polymer and the lower metal mold insert with nanocavity for different shapes, as shown in Figure 1. For the polymeric material, polypropylene (PP) was selected, and the polymerization degree of the single-chain was 20. Previous studies have shown that the polymerization degree has a significant impact on the filling capacity [35]. Nevertheless, in order to observe the separation of nanostructure from nanocavity during the demolding process, a low polymerization degree was set to allow the molecular slippage and disentanglement under the external demolding force [33]. Then, 130 chains were randomly generated to construct the amorphous polymer system with a box size of 5.0 × 5.0 × 7.9 nm. The initial density of PP was 0.92 g/cm^3^. Since the PP molecules generated in a random method had high energy, the amorphous polymer was successively optimized by energy minimization, cycle annealing, and high temperature relaxation. Consequently, each molecule of PP could eliminate part of the internal stress and continuously adjust its conformation to find the lowest energy position. Both the annealing and the relaxation temperatures of PP were 528 K.

Ni was selected as the mold insert material to investigate the influence of nanocavity shape on the non-bonded interaction at the PP-mold insert interface. The rectangular, trapezoidal, and tapered nanocavities were constructed, respectively. Because the size of the contact area directly determined the interaction energy at the interface, the interface models of different shapes with approximately equal contact area were constructed. The geometrical parameters of nanocavities are shown in Figure 1. For the study of the effect of mold insert material on the PP-mold insert interface, three materials, including Ni, silicon (Si), and the metallic oxide, Al_2_O_3_, was used. The three materials were cut into a rectangular nanocavity, and the contact area of the interface was basically equal. The periodic boundary conditions of the simulation model were selected in the X and Y directions, and the non-periodic and shrink-wrapped boundary condition was selected in the Z direction. The mold insert was regarded as a rigid body, and its position would not change throughout the whole simulation process. In order to prevent the interaction between the metal mold insert and the polymer, a vacuum layer of 2.0 nm was set above the polymer.

### 2.2. Interatomic Potential

The MD simulation of the all-atom model used the consistent valence force field (CVFF) to obtain the potential parameters for the intermolecular and intramolecular interactions [21,34,36]. The non-bonded interaction energy at the PP-mold insert interface directly determined the adhesion force, which was one of the important factors affecting the demolding quality. In the demolding process, the non-bonded interaction energy at the PP-mold insert interface, including van der Waals energy and electrostatic energy. The standard 12/6 Lennard-Jones potential and the Coulombic pairwise interaction were adopted to describe the van der Waals energy and the electrostatic interaction energy at the interface, respectively. The calculation function was given by Equation (1), as follows:

(1) $$E_{non-bonded}=E_{vdw}+E_{ele}=4\varepsilon \left[{\left(\frac{\sigma }{r}\right)}^{12}-{\left(\frac{\sigma }{r}\right)}^{6}\right]+\frac{q_{i}q_{j}}{r}(r<r_{c}),$$

where ε is a non-bonded interaction constant, σ is the distance between two atoms in equilibrium, r is the distance between two atoms at any time, $r_{c}$ is the cutoff distance, $q_{i}$ and $q_{j}$ are the charges on the two atoms. The cutoff distances for both Lennard-Jones potential and Coulombic pairwise interaction were 1.25 nm. When the distance between two atoms was greater than 12.5 nm, the non-bonded interaction energy between them will be neglected.

### 2.3. Simulation Procedure

The simulation process for MD was performed by an open-source software LAMMPS, which was distributed by Sandia National Laboratories, Livermore, CA, USA. In this study, the system temperature was jointly controlled by the NVE (constant-number, constant-volume, and constant-energy) ensemble and the Berendsen thermostat. The time step was set to 0.1 fs. In order to simplify the simulation process and improve the calculation efficiency, the filling and packing stages were performed simultaneously. PP was fully relaxed before filling the nanocavity. The force of 1.0 kcal/mol·Å (equal to 0.07 nN) along Z direction was applied to each atom of PP to complete the filling and packing stages, and a total of 50,000 steps were simulated to ensure that the nanocavity was fully filled. In this process, the temperature of PP was gradually reduced from 528 K to 393 K, while the temperature of the mold insert remained at 393 K. In the cooling process, the force applied to PP was removed, and the temperatures of both PP and mold insert were reduced from 393 K to 353 K. The whole system after cooling was used as the initial model for the demolding simulation. Then, the reverse force along -Z direction was applied to each atom of PP as the demolding force, which was also 1.0 kcal/mol·Å.

## 3. Results and Discussion

### 3.1. Influence of Nanocavity Shape

**Figure 2:**
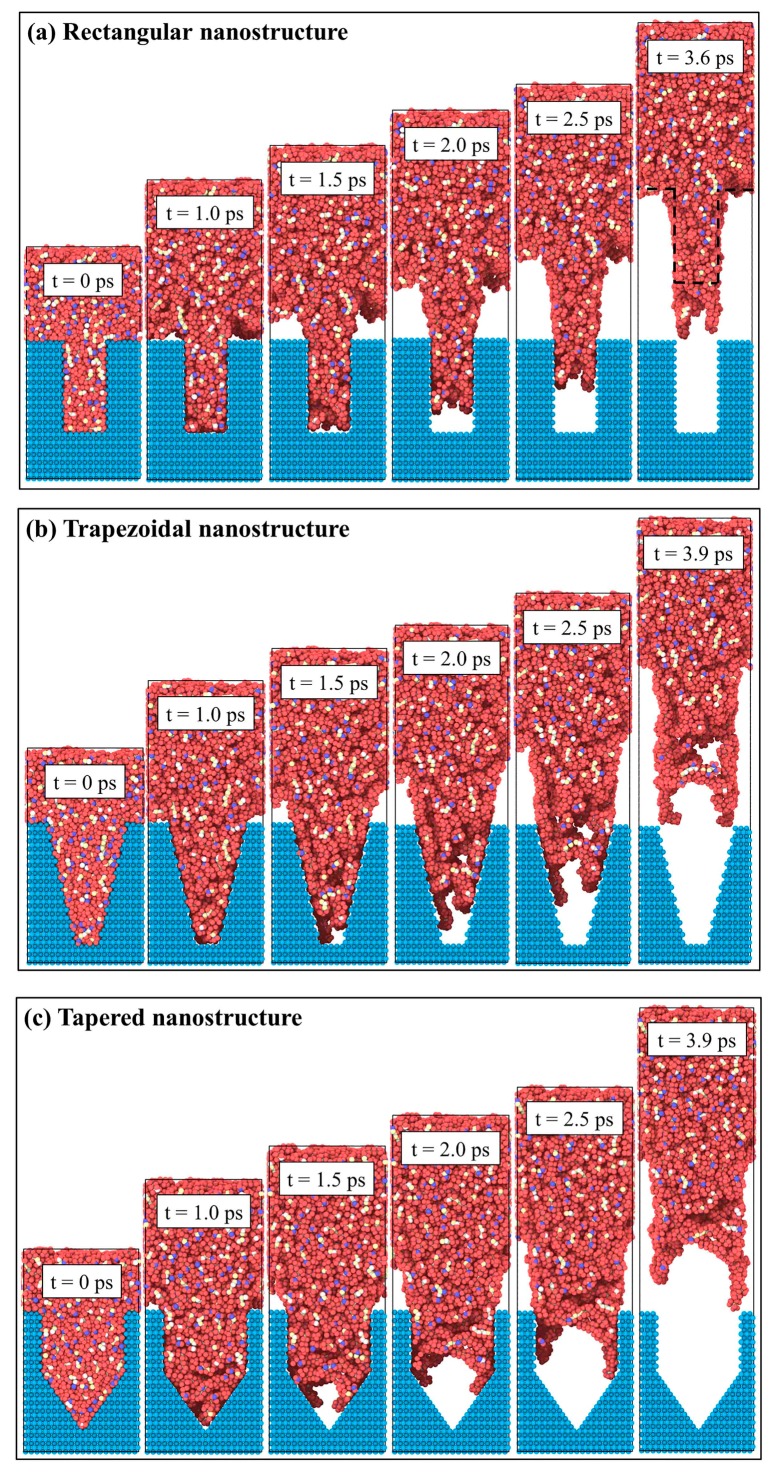
Snapshots of demolding processes for different polymer nanostructures: (**a**) Rectangular nanostructure, (**b**) trapezoidal nanostructure, (**c**) tapered nanostructure.

The MD simulation method was used to simulate the demolding process of micro-injection molding for rectangular, trapezoidal, and tapered nanocavities. In order to observe the deformation behavior of polymer nanostructures, the snapshots of demolding process are shown in Figure 2. Under the same external demolding force, the rectangular nanostructure could retain most of its original morphology after demolding, while the trapezoidal and tapered nanostructures were severely deformed, and the original morphologies were almost disappeared. There were depression deformations of different degrees at the top of nanostructures, and also many voids were inside the nanostructures. It might be due to the different specific surface areas of nanocavities, which resulted in different degrees of deformation of three nanostructures. The adhesion forces also caused three nanostructures cannot be separated from the nanocavities at 1.0 ps. After 1.0 ps, the external demolding force overcame the adhesion at the interface, and then the nanostructures began to gradually get out of the nanocavities. By observing the demolding process of the trapezoidal and tapered nanostructures, it was found that the molecular chains at the edges of nanostructures moved along the nanocavity walls, and the top of nanostructures were gradually torn. Since the rectangular nanocavity did not have the slope features of trapezoidal and tapered nanocavities, the adhesion at the PP-Ni interface would only lead to the elongation of rectangular nanostructure without tearing the structure and losing the original morphology. Consequently, the nanocavity shape had a direct impact on the demolding quality of nanostructures.

**Figure 3:**
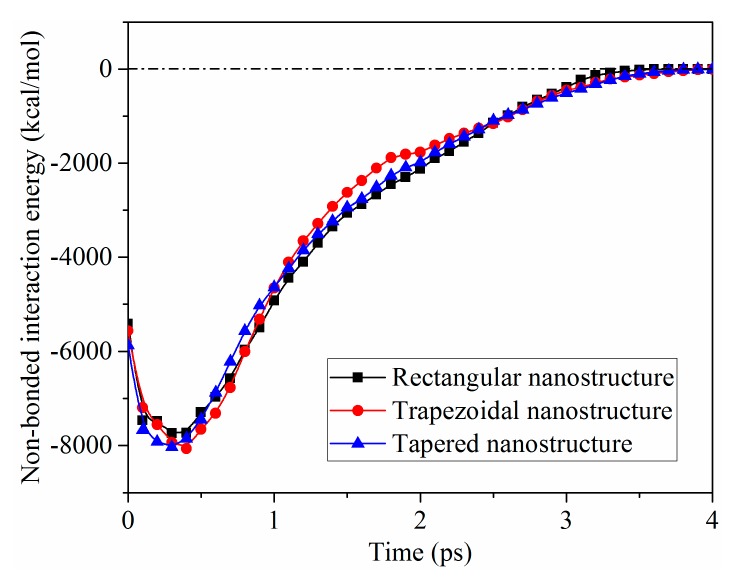
Non-bonded interaction energies of the three contact interfaces.

In order to understand the influence of nanostructure shapes on the molding quality after demolding, the equal contact areas of PP-Ni interface for different nanostructures was set. The non-bonded interaction energies at three contact interfaces were calculated, as shown in Figure 3. The value of the non-bonded interaction energies was negative, which indicated that there was a bonding between the polymer and the mold insert. The larger the absolute value was, the greater the non-bonded interaction energy was. The non-bonded interaction energies at three contact interfaces were rapidly increased at first and then gradually decreased to zero. The reason for the increase of non-bonded interaction energy at the initial moment might be that the external packing force on PP would reduce the distance between the atoms of PP and Ni during the packing process. When the distance between two atoms became very small, there was a strong repulsion between them. At the beginning of demolding, the molecular chains of PP gradually became loose, and the repulsion between the atoms gradually decreased. Consequently, the non-bonded interaction energies at the PP-Ni interfaces could be drastically increased before 0.5 ps. The maximum values of the non-bonded interaction energies of rectangular, trapezoidal, and tapered nanostructures were −7740.13, −8063.60, and −8035.03 kcal/mol, respectively. After 0.5 ps, the non-bonded interaction energies were decreased gradually with the decreasing contact area of PP-Ni interfaces. Throughout the whole demolding process, there was little difference in the non-bonded interaction energy among three contact interfaces. It could be concluded that when the contact areas were the same, the nanostructures with different shapes did not affect the non-bonded interaction energy at PP-Ni interface. The non-bonded interaction energy might greatly depend on the contact area of the PP-Ni interface.

**Figure 4:**
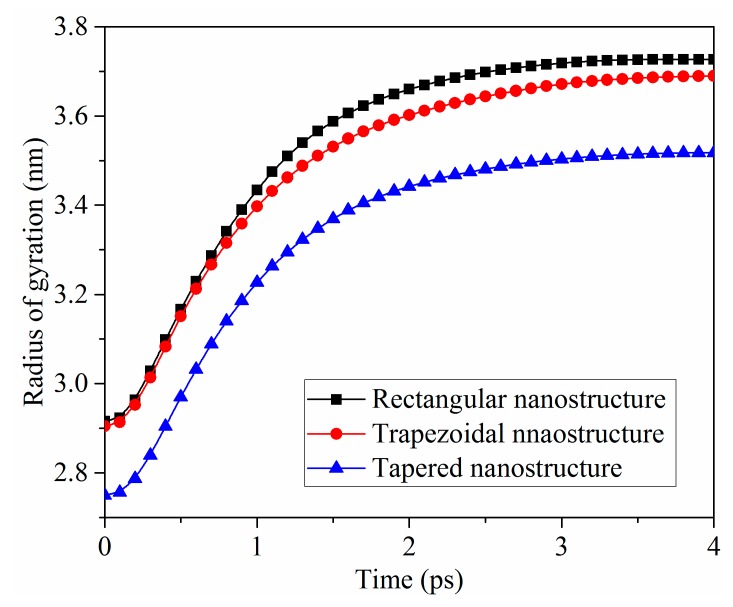
Radii of gyration of nanostructures with different shapes.

**Figure 5:**
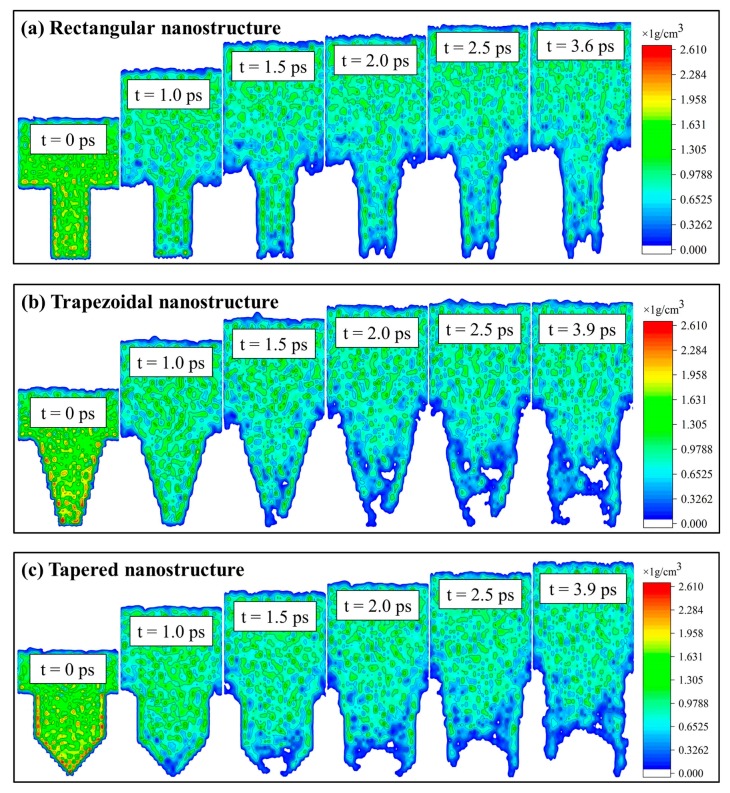
Density distributions of the demolding process of PP nanostructures with different shapes: (**a**) Rectangular nanostructure, (**b**) trapezoidal nanostructure, (**c**) tapered nanostructure.

The radii of gyration of molecular chains in PP, which are used to describe the extent of molecular chains extension in space, are shown in Figure 4. The calculation function is given by Equation (2), as follows:

(2) $$Rg^{2}=\frac{1}{M}\sum_{i}m_{i}{\left(r_{i}-r_{cm}\right)}^{2},$$

where M is the total mass of PP, $m_{i}$ is the atomic weight, $r_{cm}$ is the center of mass position of PP, $r_{i}$ is the distance of atoms to the center of mass position, and the sum is over all atoms in the whole PP. Rg is a measure of the size of the group of atoms, and is computed as the square root of the $Rg^{2}$ value in this formula. After the cooling process, the radii of gyration of rectangular, trapezoidal, and tapered nanostructures were 2.92 nm, 2.90 nm, and 2.75 nm, respectively. This might be attributed to the fact that the volume of tapered nanocavity was the largest, and the pressure in the nanocavity was also the largest. Compared with trapezoidal and rectangular nanostructures, the molecular structures of tapered nanostructure were more compact, and the distortion of molecular chains was more noticeable. Within the initial 1.0 ps, the radii of gyration of three nanostructures were increased sharply because of the adhesion with the mold insert surface. Then, as the interfacial adhesion decreased, the molecular chains were gradually changed from curled state to stretched state. Meanwhile, the radii of gyration were slowly increased to a stable value. This was consistent with the snapshots (Figure 2) and the density distributions (Figure 5) of the demolding process of PP nanostructures with different shapes.

By observing the density distributions of nanostructures, it can be seen that the polymer density near the surface area of nanocavities was much higher than that in other regions at 0 ps. This might be due to the strong non-bonded interactions with Ni atoms near the surface regions, and the molecular chains gradually accumulated near the nanocavities surface during the filling process. The densities of nanostructures decreased mainly in the early stage of demolding but did not change much in the later stage. There were many voids in nanostructures after demolding, which resulted in the densities of local areas were much lower than the average level.

### 3.2. Influence of Interfacial Adhesion Energy

When the surface of the mold insert is coated with an anti-stick coating, the nanostructure will very likely have a complete morphology after demolding. Meanwhile, defects, such as surface burrs, necking, and stretching, will significantly reduce [10,13,37]. Then the influence of interfacial adhesion on the demolding quality of nanostructure was studied. The non-bonded interaction energy at PP-Ni interface could be approximated as the adhesion energy. The value of ε for energy constant in the standard 12/6 Lennard-Jones potential was reduced to assume that the surface of nanocavity had the anti-stick coating. The values of ε were reduced by 90%, 75%, and 50%, respectively. Therefore, the non-bonded interaction energy at PP-Ni interface was correspondingly reduced.

**Figure 6:**
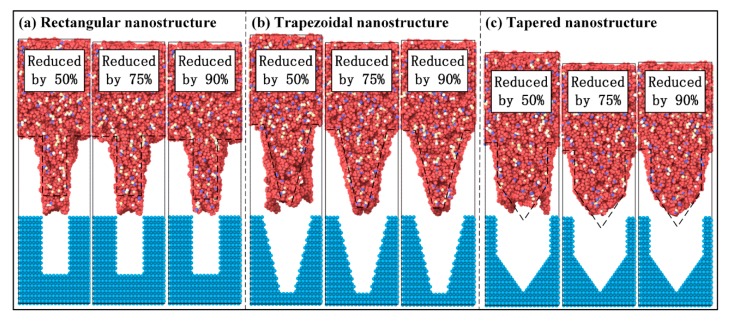
Morphologies of three nanostructures after reducing the adhesion energy at PP-Ni interface: (**a**) Rectangular nanostructure, (**b**) trapezoidal nanostructure, (**c**) tapered nanostructure.

After the adhesion energy at PP-Ni interface was reduced, the morphologies of three nanostructures after demolding are shown in Figure 6. When the adhesion energy at PP-Ni interface was reduced by 50%, the morphology of rectangular nanostructure was obviously different from that of Figure 2. Both the top depression and elongation deformations of nanostructure were greatly improved, and the structure appeared to be more compact. However, the structural characteristics of trapezoidal and tapered nanostructures were still missing after demolding. It is only that the degree of deformation at the top of nanostructures was obviously reduced. This may be due to the excessive non-bonded interaction energy at PP-Ni interface, and the strong adhesion force was still exists between the mold insert and the PP. When the adhesion energy at PP-Ni interface was reduced by 75%, the morphology of rectangular nanostructure was almost the same compared to the 50% reduction in adhesion energy. In the case of the trapezoidal and tapered nanostructures, the original features of the structures became appeared after demolding, except that the body parts were slightly enlarged. As the adhesion energy at PP-Ni interface was further reduced to 10%, the original morphologies of three nanostructures could be well preserved. Nevertheless, there was still a phenomenon of body enlargement in three nanostructures. Therefore, the demolding quality of nanostructures can be effectively improved by applying the anti-stick coating on the mold insert surface.

**Figure 7:**
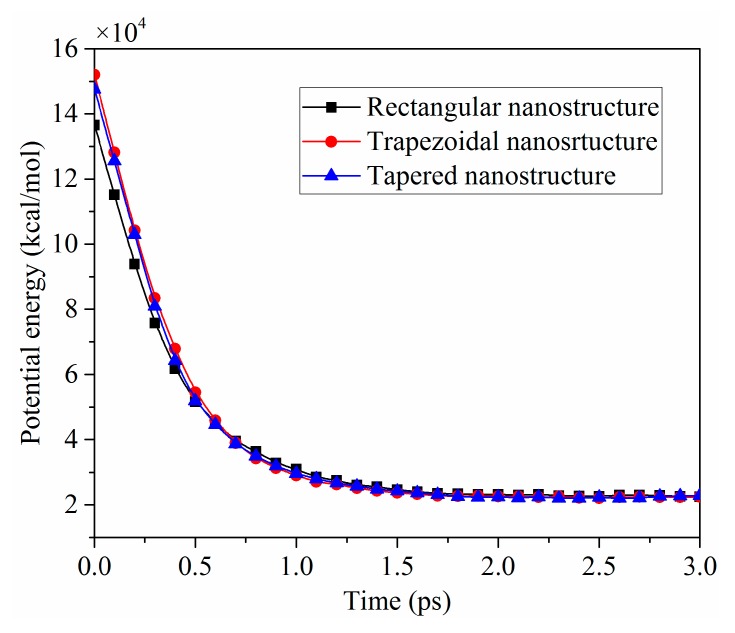
Potential energies of nanostructures with different shapes when the interfacial adhesion energies decreased by 90%.

The potential energies of three nanostructures during the demolding process were calculated when the interfacial adhesion energy decreased by 90%, as shown in Figure 7. The potential energies were produced by the interaction forces between molecules of nanostructures. In the injection and packing stages, the interaction forces between the molecules of nanostructures were increased, and the pressure was gradually stored as the potential energy. At this time, the nanostructures presented a relatively compact state. However, the potential energies were sharply released before 1.0 ps during the demolding process. With the release of the potential energy, the interactions between molecules were weakened, and the nanostructures became loose from the original state. Consequently, the body enlargement of nanostructures occurred. It is noticeable that the potential energies of the trapezoidal and tapered nanostructures were not much different, and the rectangular nanostructure was slightly lower than that both of them. This might be that the volume of rectangular nanostructure was smaller than that of the other two nanostructures. Since the changes of potential energies tended to be stable in the later stage of demolding, the deformation degree of nanostructures hardly increased. The deformations of nanostructures were not entirely attributed to the interfacial adhesion, and the release of potential energy during the demolding process was also the main reason for the deformations.

### 3.3. Influence of Mold Insert Material

**Figure 8:**
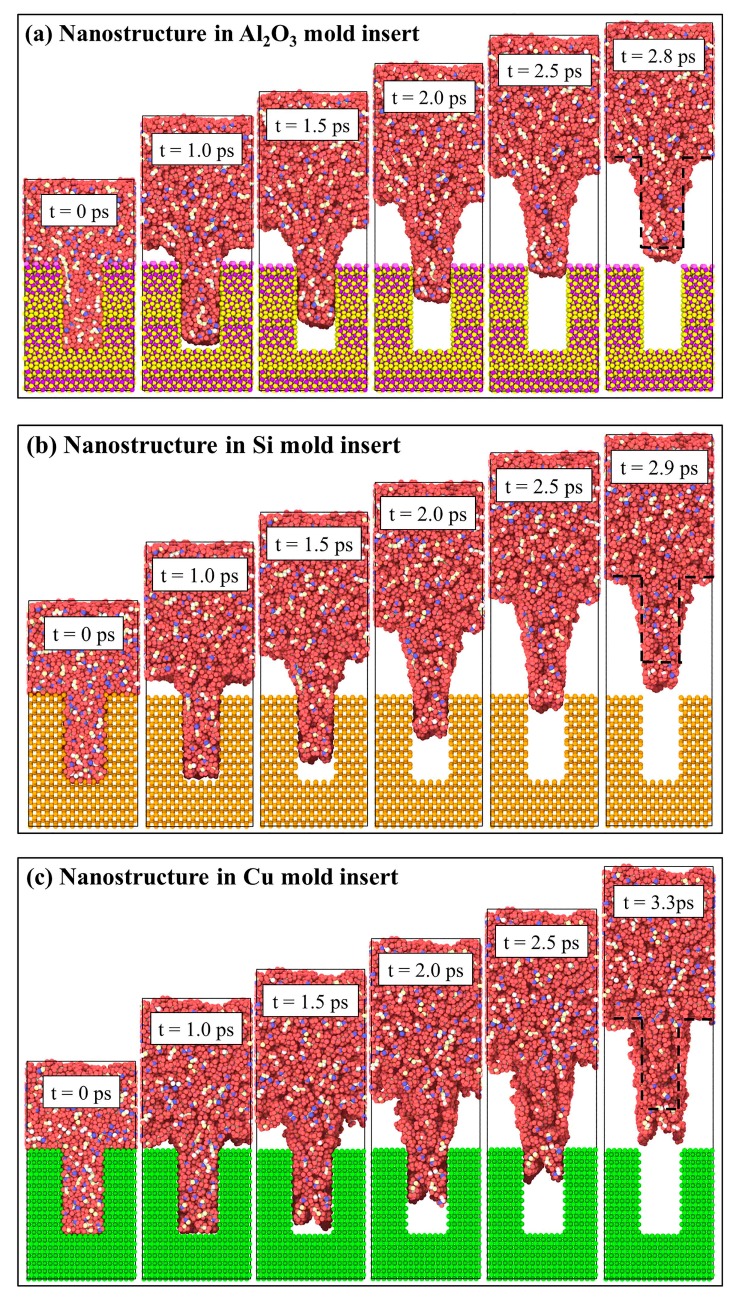
Snapshots of demolding processes of rectangular nanostructures with different mold insert materials: (**a**) Nanostructure in Al_2_O_3_ mold insert, (**b**) nanostructure in Si mold insert, (**c**) nanostructure in Cu mold insert.

Because different mold insert materials had different adhesion to PP, the influence of the common mold insert materials on the demolding quality of nanostructures was also explored in this study. The snapshots of demolding processes of rectangular nanostructures with different mold insert materials were shown in Figure 8. From the morphologies after demolding, it is clear that the nanostructures in the three mold insert materials could successfully complete demolding, but all of them had different degrees of deformation. When the mold insert material was Al_2_O_3_, the deformation of nanostructure was the smallest with slightly stretching and enlargement. It took only 2.8 ps to separate from the nanocavity completely. This might be attributed to the minimum adhesion energy at the PP-Al_2_O_3_ interface. Compared with the Ni (Figure 2) and Cu mold inserts, the time of separation from Al_2_O_3_ mold insert was greatly shortened. In the case of nanostructure in Si mold insert, its demolding quality was similar to the nanostructure in Al_2_O_3_ mold insert, but it had a larger elongation deformation. The nanostructure in Cu mold insert presented the elongation, the body enlargement, and the top depression deformations. The demolding quality was as poor as the nanostructure in Ni mold insert. The PP-Cu and PP-Ni interface might have similar adhesion properties.

**Figure 9:**
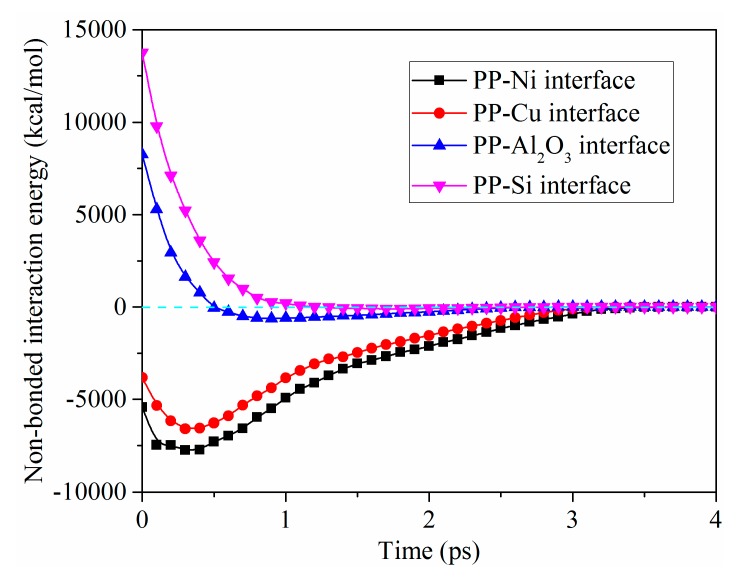
Non-bonded interaction energies of the four contact interfaces.

In order to further explain the deformation of nanostructures with different mold insert materials, the non-bonded interaction energies of the four contact interfaces were calculated, as shown in Figure 9. It can be seen that the trend of the non-bonded interaction energy at PP-Cu interface with demolding time was consistent with that of PP-Ni interface. The strong adhesion force also existed at the PP-Cu interface, which was less than that of PP-Ni interface. Therefore, the demolding quality of nanostructure in Cu mold insert was slightly better than that of Ni mold insert. As for PP-Al_2_O_3_ and PP-Si interfaces, the non-bonded interaction energies were positive in the initial stage of demolding. It showed that there was a repulsive force at the two interfaces. The repulsive force of the interfaces before 0.5 ps was released sharply, which might be one of the reasons for the bottom enlargement of nanostructures. Then the non-bonded interaction energies gradually became negative and approached zero, indicating that there was also adhesion force at the interfaces. Since the adhesion force was small enough compared to the other two interfaces, the deformation of nanostructures was much smaller than the others. The non-bonded interaction energies at PP-Ni and PP-Cu interfaces were always negative, and there was adhesion force at the two interfaces during the whole demolding process. Therefore, the deformation of nanostructures in the Ni and Cu mold inserts were the most serious.

The surface free energy and surface tension have the same dimension and value. It has been shown that the surface tension of molten PP was far less than the surface free energy of Ni, Cu, Al_2_O_3_, and Si [38,39]. Thus, the influence of PP’s surface tension on the interfacial adhesion could be negligible, and the surface free energy of the four metals would play a major role in the interfacial adhesion. According to the calculation results of the surface free energy of the four mold insert materials by some scholars, it could be concluded that the surface free energy of Ni was the maximum, followed by Cu, Al_2_O_3_, and Si was the minimum [40,41,42,43]. The surface free energy is the expression of intermolecular forces on the surface, which is closely related to the wettability of the solid surface. The smaller the surface free energy of solid material is, the better the wettability and the smaller the adhesion with polymer is. Therefore, the difference of non-bonded interaction energies of the four interfaces could be explained by the surface free energy of the mold insert materials.

**Figure 10:**
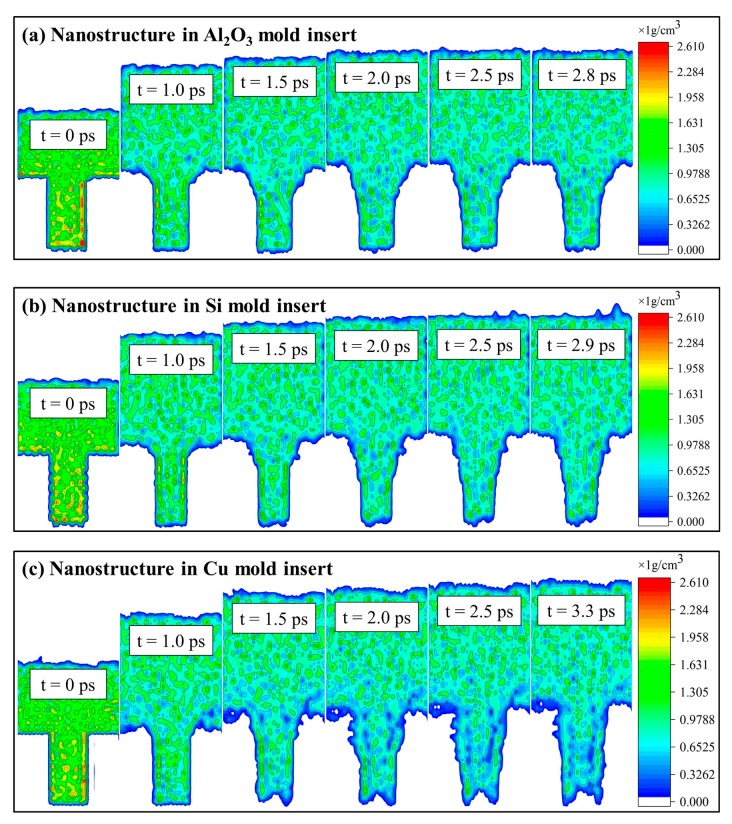
Density distributions of the demolding process of nanostructures in different mold insert materials: (**a**) Nanostructure in Al_2_O_3_ mold insert, (**b**) nanostructure in Si mold insert, (**c**) nanostructure in Cu mold insert.

The density distributions of the demolding process of nanostructures in different mold insert materials were calculated to characterize the demolding quality, as shown in Figure 10. As the same as the nanostructure in Ni mold insert, polymer molecules were likely to accumulate near the surface of the nanocavity. In the early demolding process, the density of nanostructure was decreased greatly, and many voids appeared. The molecular chains were continuously stretched by the influence of the external force as the demolding stage in progress, resulting in a continuous decrease in the density of nanostructures. When the nanostructure was completely separated from the nanocavity, it can be found that the average density of the nanostructure in the Ni mold insert was lower than the initial density of PP. However, the densities of nanostructures in Si and Al_2_O_3_ mold inserts were significantly higher than that of Ni and Cu mold insert after demolding. There was little difference in density of nanostructure between Si and Al_2_O_3_ mold materials. Consequently, the nanostructure could have good demolding quality and less structural deformation and defects when the mold materials were Al_2_O_3_ and Si.

## 4. Conclusions

In this paper, an all-atom molecular dynamics method was proposed to simulate the influence of nanostructure shape, interfacial adhesion energy, and mold insert material on demolding quality of nanostructures in micro-injection molding. The nanostructure shape had a direct impact on demolding quality. Considering roughly the same non-bonded interaction energy at PP-Ni interface, the rectangular nanostructure could keep most of its original morphology after demolding, while the trapezoidal and tapered nanostructures were severely deformed. The non-bonded interaction energy might greatly depend on the contact area of the PP-Ni interface. Due to the strong adhesion of the Ni surface, the molecular chains in nanostructures became loose, and the densities of nanostructures were decreased sharply in the early stage of demolding. The demolding quality of nanostructures can be effectively improved by adding the anti-stick coating on the mold insert surface. However, the deformations of nanostructures were not entirely attributed to the interfacial adhesion, and the release of potential energy during the demolding process was also the main reason for the body enlargement. Under the same external demolding force, the nanostructures in Al_2_O_3_ and Si mold inserts had the slightly stretching and bottom enlargement after demolding. Meanwhile, the nanostructures in Cu and Ni mold inserts appeared the elongation, the body enlargement, and the top depression deformations. The nanostructure could have good demolding quality and less structural deformation and defects when the mold materials were Al_2_O_3_ and Si. Consequently, this study would be helpful to improve the demolding quality of nanostructures.
